# Design, Synthesis, and *In Vitro* Antimicrobial Evaluation of Fused Pyrano[3,2-*e*]tetrazolo[1,5-*c*]pyrimidines and Diazepines

**DOI:** 10.1155/2013/635384

**Published:** 2013-08-21

**Authors:** Sankari Kanakaraju, P. Sagar Vijay Kumar, Bethanamudi Prasanna, G. V. P. Chandramouli

**Affiliations:** Department of Chemistry, National Institute of Technology, Warangal, Andhra Pradesh 506 004, India

## Abstract

A series of novel pyranochromene-containing tetrazoles fused with pyrimidinethiones, pyrimidines, and diazepines **3a–f**, **4a–f**, and **5a–f** were synthesized by condensation of the corresponding tetrazoles **2a–f** with carbon disulfide, benzaldehyde, and 4-methoxy phenacyl bromide, respectively. The compound **2a–f** was obtained by reaction of pyrano[3,2-*c*]chromenes **1a–f** with sodium azide. The structures of the newly synthesized compounds **2a–f** to **5a–f** were established on the basis of their elemental analyses, IR, ^1^H NMR, ^13^C NMR, and mass spectral data. All of the title compounds were subjected to *in vitro* antibacterial testing against four pathogenic strains and antifungal screening against two fungi. Preliminary results indicate that some of them exhibited promising activities and that they deserve more consideration as potential antimicrobials.

## 1. Introduction

Pyrano[3,2-*c*]chromene derivatives are a class of important heterocycles with a wide range of biological properties [1] such as spasmolytic, diuretic, anticoagulant, anticancer, and antianaphylactic activities [2–5].

Tetrazoles represent an important class of heterocyclic compounds with wide ranging applications [6]. The synthesis of novel tetrazole derivatives and the investigation of their chemical and biological behavior has gained more importance in the recent decades for biological and pharmaceutical reasons. They have found use in pharmaceuticals as lipophilic spacers and carboxylic acid surrogates, which improves oral absorption [7]. Their derivatives were reported to possess broad spectrum of biological activity in both medicinal and pharmaceutical areas such as antinociceptive [8], antibacterial [9], antifungal [10], anti-HIV, anticancer, immunosuppressive [11], anti-inflammatory [12] and antiulcer [13], antiproliferative [14], antiallergic [15], and analgesic [16] activities.

On the other hand, pyrimidine scaffold was the base of many bioactive molecules such as antitubercular, antimicrobial [17], antiviral [18], antitumor [19, 20], anti-inflammatory, analgesic [21], antioxidant [22], antiproliferative [23], and antileishmanial agents [24]. Consequently, synthetic methodologies for synthesis of novel pyrimidines or pyrimidine fused compounds are of particular interests to organic and medicinal chemists. 

The diazepine family represents one of the most prominent classes of privileged scaffolds in the field of drugs and pharmaceuticals. These compounds are widely used as anticonvulsant, antianxiety, analgesic, sedative, antidepressive, and hypnotic agents [25–27]. 

In view of the abovementioned facts, it was envisaged that these active pharmacophores, if linked together, would generate novel molecular templates which are likely to exhibit interesting biological properties. Hence, in continuation of our interest in the synthesis of biologically active heterocycles [28, 29], we report herein the synthesis and antimicrobial evaluation of some new heterocyclic compounds like pyranochromenes-containing tetrazoles. These tetrazoles are fused to six and seven-membered heterocyclic rings like pyrimidines, pyrimidinethiones, and diazepines. This combination was suggested in an attempt to investigate the influence of such structure variation on the anticipated biological activities, hoping to add some synergistic biological significance to the target molecules.

## 2. Results and Discussion

**Scheme 1:**
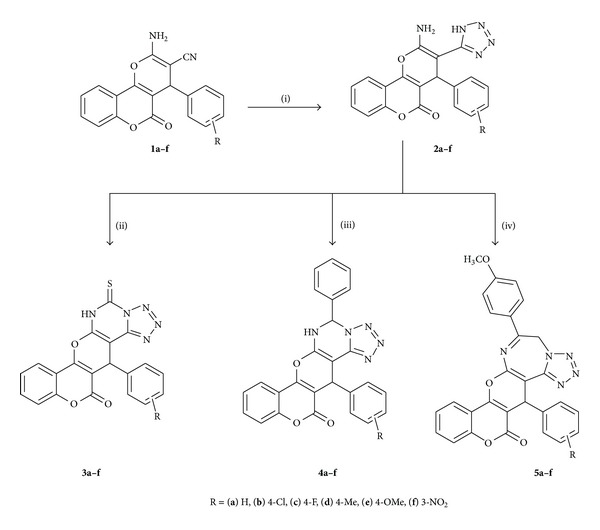
Schematic representation for the synthesis of fused tetrazole derivatives. Reagents and conditions: (i) NaN_3_, DMF, NH_4_Cl, 120°C, 7 h; (ii) CS_2_, pyridine, reflux, 10 h; (iii) conc. HCl, benzaldehyde, MeOH, reflux, 16 h; (iv) 4-methoxyphenacyl bromide, EtOH, AcONa, reflux, 16 h.

The synthetic strategies adopted for the synthesis of the target compounds are depicted in Scheme 1. The starting materials used in the present scheme, that is, pyrano[3,2-*c*]chromenes **1a–f**, were prepared according to the previously reported procedure [30]. Pyrano[3,2-*c*]chromenes **1a–f** were allowed to react with sodium azide in presence of NH_4_Cl in DMF to give the corresponding tetrazoles **2a–f**, which on reaction with CS_2_ in presence of pyridine yielded the corresponding pyrimidine-5-thiones **3a–f**. Condensation of tetrazoles **2a–f** with benzaldehyde furnished the respective pyrimidines **4a–f**. The compounds **5a–f** were obtained by condensing tetrazoles **2a–f** with 4-methoxyphenacyl bromide under reflux in ethanol. The structures of all of the newly synthesized compounds were elucidated on the basis of their spectral (IR, ^1^H NMR, ^13^C NMR, and mass) and elemental analyses data. The synthesized compounds **2a–f**, **3a–f**, **4a–f**, and **5a–f** were also assayed for their antimicrobial activities.

The formation of tetrazoles **2a–f** from pyrano[3,2-*c*]chromenes **1a–f** was confirmed by their IR, ^1^H NMR, and ^13^C NMR. In IR spectrum the disappearance of sharp absorption band (–CN) around 2200 cm^−1^ and the appearance of band (–NH) around 3300 cm^−1^ showed the formation of tetrazole, while in ^1^H NMR the tetrazole NH proton was observed as a singlet at *δ* 10.01–10.21 ppm and in ^13^C NMR the tetrazole carbon was observed around *δ* 159.18–159.82 ppm. Furthermore, the condensation product **3a–f** was confirmed by its IR spectrum which showed absence of the characteristic absorption band due to the –NH_2_ group, and according to the IR spectroscopic data of these **3a–f** compounds which have tetrazolopyrimidin-5-thione structure, the observation of the C=S stretching band at 1165–1181 cm^−1^, and the absence of an absorption band at about 2550–2600 cm^−1^ region cited for SH group have proved that these compounds were in the thionic form. In their ^13^C NMR, peak due to C=S appeared at *δ* 189.72–190.16 ppm. The formation of **4a–f** from **2a–f** was confirmed by their IR spectra in which no –NH_2_ stretching vibrations were observed, and were well supported by their ^1^H NMR and ^13^C NMR. In ^1^H NMR, peaks shown at *δ* 6.22–6.34 ppm were assigned to pyrimidine CH proton, and in ^13^C NMR the signal was observed at *δ* 63.51–64.38 ppm due to pyrimidine carbon. Other signals were found in accordance to the established structures. Furthermore, the conversion of tetrazoles **2a–f** to fused tetrazolodiazepines **5a–f** was also well confirmed by their IR, ^1^H NMR, and ^13^C NMR spectra. The disappearance of characteristic peaks due to NH_2_ and NH groups of **2a–f** clearly indicated the smooth cyclization. Their ^1^H NMR spectra showed a singlet at *δ* 6.13–6.32 ppm for two protons, which is attributed to the methylene protons of diazepine ring, while in ^13^C NMR the signal that appeared at *δ* 48.43–49.06 ppm was assigned to methylene carbon of diazepine ring. For all of the compounds, the band displayed at 1700–1719 cm^−1^ was due to the lactone carbonyl group, which was observed around *δ* 160.22–161.93 ppm in ^13^C NMR. The signal due to pyran proton, present in all of the compounds, appeared as a singlet at *δ* 4.56–5.12 ppm. All of the other aromatic and aliphatic protons were observed at the expected regions. Mass spectra of almost all of the compounds showed M + 1 peaks, in agreement with their molecular weights. However, in some cases, M + 2 peaks were also observed. For all of the compounds, the elemental analyses values were in good agreement with the theoretical data. Full characterization details were provided in the Experimental section.

### 2.1. Antimicrobial Activity

The antibacterial activity of the synthesized compounds **2a–f**,** 3a–f**,** 4a–f**, and **5a–f** was screened against the Gram-positive bacteria such as *Bacillus subtilis* and* Staphylococcus aureus* and the Gram-negative bacteria, that is,* Pseudomonas aeruginosa* and *Escherichia coli* using nutrient agar medium. The antifungal activity of the compounds was tested against *Candia albicans *and *Aspergillus niger *using Potato dextrose agar (PDA) medium. The minimum inhibitory concentration (MIC) was carried out using microdilution susceptibility method [31]. Ciprofloxacin was used as a standard antibacterial drug, and fluconazole was used as a standard antifungal drug. The observed data on the antimicrobial activity of compounds and control drugs are given in Table 1.

The MIC values were determined as the lowest concentration that completely inhibited visible growth of the microorganisms. The investigation of antibacterial screening (Table 1) revealed that some of the newly synthesized compounds showed moderate-to-good inhibition at 25–100 *μ*g/mL in DMSO. Amongst all of the compounds, compounds **3b** and **3e** exhibited good activities against *B. subtilis* (MIC: 25 *μ*g/mL) and *E. coli* (MIC: 50 and 25 *μ*g/mL) and moderate activities against *S. aureus* and *P. aeruginosa*. Compound **4b** displayed good activity against *S. aureus* (MIC: 50 *μ*g/mL), whereas compound **4e** exhibited good activity against *P. aeuroginosa* (MIC: 50 *μ*g/mL).

The investigation of antifungal screening (Table 1) revealed that some of the newly synthesized compounds showed moderate-to-good inhibition at 25–100 *μ*g/mL in DMSO. Among the tested compounds, compounds **2b**, **3b**, and **4e** were found to be more active than other compounds against *A. niger* (MIC: 50 *μ*g/mL). Compounds **2c**, **3e**, and **4b** possess good activities against *C. albicans* (MIC: 50 *μ*g/mL). Compounds **2f**, **5a**, and **5f** showed no activity against the tested species. Remaining compounds showed moderate-to-least activity against both bacteria and fungi. The structure activity relationship of the synthesized compounds revealed that the compounds having diazepine ring showed the least activity compared with other compounds. The presence of chloro and methoxy group enhances the activities of the compounds. The maximum antimicrobial activity was observed with compounds **3b**, **3e**, **4b**, and **4e**.

## 3. Conclusion

In summary, a series of novel pyranochromene-containing fused tetrazole derivatives have been synthesized and characterized by spectral and elemental analyses. All of the newly synthesized compounds were screened for their *in vitro* antimicrobial activities. Among the screened samples, **3b**, **3e**, **4b**, and **4e** showed significant antibacterial and antifungal activities compared with other tested samples.

## 4. Experimental

Melting points were recorded in open capillary and were uncorrected. Column chromatography was performed using silica-gel (100–200 mesh size) purchased from Thomas Baker, and thin-layer chromatography (TLC) was carried out using aluminium sheets precoated with silica-gel 60F_254_ purchased from Merck. IR spectra (KBr) were obtained using a Bruker WM-4(X) spectrometer (577 model). ^1^H NMR (400 MHz) and ^13^C NMR (100 MHz) spectra were recorded on a Bruker WM-400 spectrometer in DMSO-*d*_6_ with TMS as an internal standard. Mass spectra (ESI) were carried out on a JEOL SX-102 spectrometer. CHN analysis was done by the Carlo Erba EA 1108 automatic elemental analyzer. The chemicals and solvents used were of commercial grade and were used without further purification unless otherwise stated.

### 4.1. General Procedure for the Synthesis of 2-Amino-4-aryl-3-(1*H*-tetrazol-5-yl)pyrano[3,2-*c*]chromen-5(4*H*)-ones **(2a–f)**

To a mixture of compound **1a–f** (10 mmol) in DMF (25 mL), sodium azide (0.78 g, 12 mmol) and NH_4_Cl (0.64 g, 12 mmol) were added, and the reaction mixture was stirred at 120°C for 7 h. The progress of the reaction was monitored by TLC. After completion of the reaction, the reaction mixture was cooled to room temperature and poured into ice cold water (100 mL). The solid separated was filtered, washed with water, dried, and purified by column chromatography on silica-gel using hexane/ethyl acetate (7 : 3) as eluent to afford compound **2a–f**.

### 4.2. 2-Amino-4-phenyl-3-(1*H*-tetrazol-5-yl)pyrano[3,2-*c*]chromen-5(4*H*)-one **(2a)**

White solid, yield 2.58 g (72%), m.p. 213–216°C; IR (KBr, cm^−1^): 3386, 3278, 3196, 2992, 1706, 1660, 1567, 1497, 1310, 1279, 1110, 1051; ^1^H NMR (400 MHz, DMSO-*d*_6_): *δ* 4.74 (s, 1H, H-4), 7.24–7.34 (m, 5H, ArH), 7.41 (brs, 2H, NH_2_), 7.45–7.52 (m, 2H, ArH), 7.69–7.89 (m, 1H, ArH), 7.91 (d, 1H, ArH), 10.01 (s, 1H, NH); ^13^C NMR (100 MHz, DMSO-*d*_6_): *δ* 36.96, 89.03, 103.97, 112.93, 119.21, 122.43, 124.62, 127.09, 127.61, 128.49, 132.87, 143.31, 152.10, 153.38, 157.97, 159.50, 161.10; MS *m/z*: 360 (M+1)^+^. Anal. Calcd for C_19_H_13_N_5_O_3_: C, 63.51; H, 3.65; N, 19.49. Found: C, 63.62; H, 3.71; N, 19.38 %.

### 4.3. 2-Amino-4-(4-chlorophenyl)-3-(1*H*-tetrazol-5-yl)pyrano[3,2-c]chromen-5(4*H*)-one **(2b)**

White solid, yield 2.67 g (68%), m.p. 192–194°C; IR (KBr, cm^−1^): 3397, 3284, 3193, 2990, 1713, 1664, 1551, 1493, 1321, 1281, 1121, 1063; ^1^H NMR (400 MHz, DMSO-*d*_6_): *δ* 4.66 (s, 1H, H-4), 7.30 (d, 2H, ArH), 7.36–7.44 (m, 4H, ArH + NH_2_), 7.48 (d, 1H, ArH), 7.54 (t, 1H, ArH), 7.78 (t, 1H, ArH), 7.92 (d, 1H, ArH), 10.14 (s, 1H, NH); ^13^C NMR (100 MHz, DMSO-*d*_6_): *δ* 37.12, 88.12, 103.89, 113.88, 119.73, 123.34, 125.48, 129.32, 130.51, 132.66, 133.71, 143.15, 153.14, 154.41, 158.13, 159.64, 160.25; MS *m/z*: 394 (M+1)^+^. Anal. Calcd for C_19_H_12_ClN_5_O_3_: C, 57.95; H, 3.07; N, 17.78. Found: C, 57.83; H, 3.14; N, 17.86%.

### 4.4. 2-Amino-4-(4-fluorophenyl)-3-(1*H*-tetrazol-5-yl)pyrano[3,2-*c*]chromen-5(4*H*)-one **(2c)**

Brown solid, yield 2.26 g (60%), m.p. 161–164°C; IR (KBr, cm^−1^): 3416, 3378, 3296, 2998, 1719, 1666, 1566, 1491, 1322, 1284, 1114, 1053; ^1^H NMR (400 MHz, DMSO-*d*_6_): *δ* 4.62 (s, 1H, H-4), 7.28 (d, 2H, ArH), 7.34–7.46 (m, 4H, ArH + NH_2_), 7.50 (d, 1H, ArH), 7.58 (t, 1H, ArH), 7.75 (t, 1H, ArH), 7.94 (d, 1H, ArH), 10.06 (s, 1H, NH); ^13^C NMR (100 MHz, DMSO-*d*_6_): *δ* 38.17, 88.43, 103.72, 112.67, 120.14, 123.42, 125.36, 129.12, 130.64, 132.48, 133.52, 143.76, 153.96, 154.71, 158.25, 159.73, 160.41; MS *m/z*: 378 (M+1)^+^. Anal. Calcd for C_19_H_12_FN_5_O_3_: C, 60.48; H, 3.21; N, 18.56. Found: C, 60.59; H, 3.26; N, 18.43%.

### 4.5. 2-Amino-3-(1*H*-tetrazol-5-yl)-4-*p*-tolylpyrano[3,2-*c*]chromen-5(4*H*)-one **(2d)**

White solid, yield 2.34 g (63%); m.p. 208–210°C; IR (KBr, cm^−1^): 3392, 3287, 3206, 2982, 1708, 1661, 1555, 1494, 1319, 1273, 1125, 1057; ^1^H NMR (400 MHz, DMSO-*d*_6_): *δ* 2.38 (s, 3H, CH_3_), 4.63 (s, 1H, H-4), 7.24 (d, 2H, ArH), 7.32–7.40 (m, 4H, ArH + NH_2_), 7.48 (d, 1H, ArH), 7.54 (t, 1H, ArH), 7.71 (t, 1H, ArH), 7.84 (d, 1H, ArH), 10.02 (s, 1H, NH); ^13^C NMR (100 MHz, DMSO-*d*_6_): *δ* 20.94, 37.62, 88.26, 103.21, 118.83, 119.76, 122.48, 125.28, 129.32, 130.61, 131.56, 133.68, 142.64, 153.06, 154.24, 158.41, 159.18, 160.22; MS *m/z*: 374 (M+1)^+^. Anal. Calcd for C_20_H_15_N_5_O_3_: C, 64.34; H, 4.05; N, 18.76. Found: C, 64.46; H, 4.11; N, 18.62%.

### 4.6. 2-Amino-4-(4-methoxyphenyl)-3-(1*H*-tetrazol-5-yl)pyrano[3,2-*c*]chromen-5(4*H*)-one **(2e)**

Orange solid, yield 2.95 g (76%); m.p. 231–233°C; IR (KBr, cm^−1^): 3419, 3376, 3284, 2992, 1716, 1668, 1566, 1492, 1320, 1283, 1117, 1061; ^1^H NMR (400 MHz, DMSO-*d*_6_): *δ* 3.84 (s, 3H, OCH_3_), 4.71 (s, 1H, H-4), 6.92 (d, 2H, ArH), 7.18–7.34 (m, 4H, ArH + NH_2_), 7.45 (d, 1H, ArH), 7.51 (t, 1H, ArH), 7.70 (t, 1H, ArH), 7.89 (d, 1H, ArH), 10.16 (s, 1H, NH); ^13^C NMR (100 MHz, DMSO-*d*_6_): *δ* 38.84, 55.90, 89.24, 105.13, 113.72, 114.81, 120.12, 123.26, 125.43, 129.52, 133.55, 136.17, 152.94, 153.82, 157.56, 158.24, 159.72, 160.36; MS *m/z*: 390 (M+1)^+^. Anal. Calcd for C_20_H_15_N_5_O_4_: C, 61.69; H, 3.88; N, 17.99. Found: C, 61.74; H, 3.77; N, 17.87%.

### 4.7. 2-Amino-4-(3-nitrophenyl)-3-(1*H*-tetrazol-5-yl)pyrano[3,2-*c*]chromen-5(4*H*)-one **(2f)**

White solid, yield 2.26 g (56%); m.p. 198–200°C; IR (KBr, cm^−1^): 3408, 3388, 3294, 2990, 1718, 1663, 1561, 1495, 1318, 1264, 1122, 1051; ^1^H NMR (400 MHz, DMSO-*d*_6_): *δ* 4.92 (s, 1H, H-4), 7.42–7.52 (m, 4H, ArH + NH_2_), 7.64 (t, 1H, ArH), 7.73 (t, 1H, ArH), 7.84 (d, 1H, ArH), 7.92 (d, 1H, ArH), 8.12 (d, 1H, ArH), 8.16 (s, 1H, ArH), 10.21 (s, 1H, NH); ^13^C NMR (100 MHz, DMSO-*d*_6_): *δ* 38.92, 89.43, 103.82, 113.71, 119.62, 123.11, 123.23, 123.64, 125.46, 130.29, 133.69, 135.36, 146.61, 148.27, 153.13, 154.56, 158.81, 159.82, 160.74; MS *m/z*: 405 (M+1)^+^. Anal. Calcd for C_19_H_12_N_6_O_5_: C, 56.44; H, 2.99; N, 20.78. Found: C, 56.50; H, 3.08; N, 20.67%.

### 4.8. General Procedure for the Synthesis of Fused Pyrano[3,2-*e*]tetrazolo[1,5-*c*]pyrimidin-5-thiones **(3a–f)**

A mixture of compound **2a–f** (2 mmol) and carbon disulfide (0.15 g, 0.12 mL, 2 mmol) in pyridine (10 mL) was refluxed on a water bath for 10 h. After completion of the reaction (monitored by TLC), the reaction mixture was cooled to room temperature, then poured into ice cold water (20 mL), and neutralized with hydrochloric acid (1 : 1). The solid obtained was filtered, washed with water, dried, and recrystallized from ethanol to afford compound **3a–f**.

### 4.9. 14-Phenyl-5,6-dihydro-13-oxo-5*H*,13*H*,14*H*-[1]benzopyrano[3′,4′:5,6]pyrano[3,2-*e*][1,2,3,4]tetrazolo[1,5-*c*]pyrimidin-5-thione **(3a)**

White solid, yield 0.54 g (68%); m.p. 226–228°C; IR (KBr, cm^−1^): 3399, 2990, 1710, 1664, 1598, 1505, 1310, 1261, 1181, 1089; ^1^H NMR (400 MHz, DMSO-*d*_6_): *δ* 5.03 (s, 1H, H-4), 7.09–7.57 (m, 7H, ArH), 7.87–7.95 (m, 2H, ArH), 10.09 (s, 1H, NH); ^13^C NMR (100 MHz, DMSO-*d*_6_): *δ* 32.45, 86.53, 104.19, 118.14, 123.63, 124.25, 126.65, 127.99, 128.09, 132.02, 139.88, 144.59, 152.11, 155.59, 156.08, 160.12, 161.93, 190.02; MS *m/z*: 403 (M+2)^+^. Anal. Calcd for C_20_H_11_N_5_O_3_S: C, 59.84; H, 2.76; N, 17.45. Found: C, 59.88; H, 2.85; N, 17.34%.

### 4.10. 14-(4-Chlorophenyl)-5,6-dihydro-13-oxo-5*H*,13*H*,14*H*-[1]benzopyrano[3′,4′:5,6]pyrano[3,2-*e*][1,2,3,4]tetrazolo[1,5-*c*]pyrimidin-5-thione **(3b)**

White solid, yield 0.57 g (66%); m.p. 185–188°C; IR (KBr, cm^−1^): 3406, 2998, 1718, 1668, 1583, 1508, 1323, 1267, 1177, 1082; ^1^H NMR (400 MHz, DMSO-*d*_6_): *δ* 4.76 (s, 1H, H-4), 7.28 (d, 2H, ArH), 7.37 (d, 2H, ArH), 7.51 (d, 1H, ArH), 7.56 (t, 1H, ArH), 7.77 (t, 1H, ArH), 7.95 (d, 1H, ArH), 10.21 (s, 1H, NH); ^13^C NMR (100 MHz, DMSO-*d*_6_): *δ* 33.44, 86.17, 103.74, 113.42, 119.76, 123.29, 124.82, 129.46, 130.32, 132.71, 133.54, 142.88, 152.96, 153.88, 157.12, 160.47, 161.12, 189.82; MS *m/z*: 436 (M+1)^+^. Anal. Calcd for C_20_H_10_ClN_5_O_3_S: C, 55.11; H, 2.31; N, 16.07. Found: C, 55.16; H, 2.23; N, 16.15%.

### 4.11. 14-(4-Fluorophenyl)-5,6-dihydro-13-oxo-5*H*,13*H*,14*H*-[1]benzopyrano[3′,4′:5,6]pyrano[3,2-*e*][1,2,3,4]tetrazolo[1,5-*c*]pyrimidin-5-thione **(3c)**

White solid, yield 0.52 (63%); m.p. 173–176°C; IR (KBr, cm^−1^): 3386, 2985, 1712, 1666, 1577, 1512, 1321, 1256, 1167, 1076 cm^−1^; ^1^H NMR (400 MHz, DMSO-*d*_6_): *δ* 4.84 (s, 1H, H-4), 7.36 (d, 2H, ArH), 7.48 (d, 2H, ArH), 7.54 (d, 1H, ArH), 7.62–7.74 (m, 2H, ArH), 7.94 (d, 1H, ArH), 10.34 (s, 1H, NH); ^13^C NMR (100 MHz, DMSO-*d*_6_): *δ* 34.21, 87.34, 103.48, 112.56, 120.71, 123.56, 124.22, 128.96, 130.82, 132.45, 133.68, 143.18, 153.27, 154.40, 157.57, 160.19, 161.56, 189.93; MS *m/z*: 421 (M+2)^+^. Anal. Calcd for C_20_H_10_FN_5_O_3_S: C, 57.28; H, 2.40; N, 16.70. Found: C, 57.39; H, 2.47; N, 16.62%.

### 4.12. 14-(4-Methylphenyl)-5,6-dihydro-13-oxo-5*H*,13*H*,14*H*-[1]benzopyrano[3′,4′:5,6]-pyrano[3,2-*e*][1,2,3,4]tetrazolo[1,5-*c*]pyrimidin-5-thione **(3d)**

White solid, yield 0.49 g (60%); m.p. 190–192°C; IR (KBr, cm^−1^): 3411, 2982, 1700, 1661, 1592, 1507, 1313, 1266, 1172, 1083; ^1^H NMR (400 MHz, DMSO-*d*_6_): *δ* 2.30 (s, 3H, CH_3_), 4.91 (s, 1H, H-4), 7.12 (d, 2H, ArH), 7.38 (d, 2H, ArH), 7.45 (d, 1H, ArH), 7.52–7.79 (m, 2H, ArH), 7.91 (d, 1H, ArH), 10.28 (s, 1H, NH); ^13^C NMR (100 MHz, DMSO-*d*_6_): *δ* 20.96, 34.52, 87.64, 103.18, 118.36, 119.82, 122.57, 125.39, 125.43, 130.72, 131.44, 133.84, 142.72, 153.14, 154.36, 158.74, 159.72, 161.78, 189.72; MS *m/z*: 416 (M+1)^+^. Anal. Calcd for C_21_H_13_N_5_O_3_S: C, 60.71; H, 3.15; N, 16.86. Found: C, 60.84; H, 3.20; N, 16.75%.

### 4.13. 14-(4-Methoxyphenyl)-5,6-dihydro-13-oxo-5*H*,13*H*,14*H*-[1]benzopyrano[3′,4′:5,6]pyrano[3,2-*e*][1,2,3,4]tetrazolo[1,5-*c*]pyrimidin-5-thione **(3e)**

White solid, yield 0.50 g (59%); m.p. 200–203°C; IR (KBr, cm^−1^): 3393, 2994, 1711, 1653, 1588, 1503, 1322, 1268, 1165, 1081; ^1^H NMR (400 MHz, DMSO-*d*_6_): *δ* 3.86 (s, 3H, OCH_3_), 4.93 (s, 1H, H-4), 6.98 (d, 2H, ArH), 7.22 (d, 2H, ArH), 7.48 (d, 1H, ArH), 7.54 (t, 1H, ArH), 7.76 (t, 1H, ArH), 7.89 (d, 1H, ArH), 10.33 (s, 1H, NH); ^13^C NMR (100 MHz, DMSO-*d*_6_): *δ* 34.64, 55.96, 88.71, 105.24, 113.64, 114.78, 120.22, 123.64, 125.49, 129.31, 133.57, 136.23, 152.88, 153.91, 158.71, 159.76, 159.88, 161.28, 189.83; MS *m/z*: 432 (M+1)^+^. Anal. Calcd for C_21_H_13_N_5_O_4_S: C, 58.46; H, 3.04; N, 16.23. Found: C, 58.39; H, 3.14; N, 16.32%.

### 4.14. 14-(3-Nitrophenyl)-5,6-dihydro-13-oxo-5*H*,13*H*,14*H*-[1]benzopyrano[3′,4′:5,6]pyrano[3,2-*e*][1,2,3,4]tetrazolo[1,5-*c*]pyrimidin-5-thione **(3f)**

White solid, yield 0.45 g (51%); m.p. 211–213°C; IR (KBr, cm^−1^): 3396, 2997, 1716, 1666, 1591, 1511, 1314, 1266, 1180, 1079; ^1^H NMR (400 MHz, DMSO-*d*_6_): *δ* 5.10 (s, 1H, H-4), 7.44 (d, 1H, ArH), 7.51 (t, 1H, ArH), 7.62 (t, 1H, ArH), 7.75 (t, 1H, ArH), 7.82 (d, 1H, ArH), 7.94 (d, 1H, ArH), 8.10 (d, 1H, ArH), 8.18 (s, 1H, ArH), 10.48 (s, 1H, NH); ^13^C NMR (100 MHz, DMSO-*d*_6_): *δ* 34.79, 88.76, 103.93, 113.62, 119.84, 123.32, 123.43, 123.79, 125.62, 130.71, 133.82, 135.43, 146.67, 148.54, 153.12, 155.93, 159.72, 160.74, 161.87, 190.16; MS *m/z*: 447 (M+1)^+^. Anal. Calcd for C_20_H_10_N_6_O_5_S: C, 53.81; H, 2.26; N, 18.83. Found: C, 53.92; H, 2.35; N, 18.78%.

### 4.15. General Procedure for the Synthesis of Fused Pyrano[3,2-*e*]tetrazolo[1,5-*c*]pyrimidines **(4a–f)**

To a mixture of compound **2a–f** (2 mmol) and benzaldehyde (0.212 g, 0.20 mL, 2 mmol) in methanol (10 mL), conc. HCl (0.5 mL) was added, and the reaction mixture was refluxed for 16 h. The progress of the reaction was monitored by TLC. After completion of the reaction, the reaction mixture was cooled to room temperature and neutralized with saturated sodium bicarbonate solution; the solid separated was filtered, washed with water, dried, and recrystallized from ethanol to afford compound **4a–f**.

### 4.16. 5,14-Diphenyl-6-hydro-13-oxo-5*H*,13*H*,14*H*-[1]benzopyrano[3′,4′:5,6]pyrano[3,2-*e*][1,2,3,4]tetrazolo[1,5-*c*]pyrimidine **(4a)**

White solid, yield 0.54 g (61%); m.p. 168–170°C; IR (KBr, cm^−1^): 3384, 2997, 1714, 1635, 1593, 1368, 1261, 1123; ^1^H NMR (400 MHz, DMSO-*d*_6_): *δ* 4.58 (s, 1H, H-4), 6.32 (s, 1H, CH), 7.14–7.58 (m, 11H, ArH), 7.82–7.86 (m, 2H, ArH), 7.94 (d, 1H, ArH), 9.60 (s, 1H, NH); ^13^C NMR (100 MHz, DMSO-*d*_6_): *δ* 35.96, 63.51, 88.32, 103.86, 113.74, 118.44, 123.30, 123.41, 123.90, 124.00, 125.27, 126.63, 127.87, 131.53, 131.85, 140.57, 143.77, 152.24, 153.69, 156.82, 159.68, 160.36; MS *m/z*: 448 (M+1)^+^. Anal. Calcd for C_26_H_17_N_5_O_3_: C, 69.79; H, 3.83; N, 15.65. Found: C, 69.72; H, 3.92; N, 15.57%.

### 4.17. 14-(4-Chlorophenyl)-5-phenyl-6-hydro-13-oxo-5*H*,13*H*,14*H*-[1]benzopyrano[3′,4′:5,6]pyrano[3,2-*e*][1,2,3,4]tetrazolo[1,5-*c*]pyrimidine **(4b)**

White solid, yield 0.51 g (53%); m.p. 155–158°C; IR (KBr, cm^−1^): 3391, 2982, 1717, 1665, 1571, 1372, 1258, 1132; ^1^H NMR (400 MHz, DMSO-*d*_6_): *δ* 4.78 (s, 1H, H-4), 6.24 (s, 1H, CH), 7.10–7.46 (m, 9H, ArH), 7.52 (d, 1H, ArH), 7.58 (t, 1H, ArH), 7.79 (t, 1H, ArH), 7.94 (d, 1H, ArH), 9.93 (s, 1H, NH); ^13^C NMR (100 MHz, DMSO-*d*_6_): *δ* 36.24, 63.72, 88.41, 104.76, 114.54, 118.73, 124.44, 125.59, 126.68, 127.82, 129.46, 130.28, 131.47, 132.61, 133.48, 138.12, 142.23, 153.17, 154.53, 156.22, 159.83, 160.48; MS *m/z*: 482 (M+1)^+^. Anal. Calcd for C_26_H_16_ClN_5_O_3_: C, 64.80; H, 3.35; N, 14.53. Found: C, 64.86; H, 3.46; N, 14.44%.

### 4.18. 14-(4-Fluorophenyl)-5-phenyl-6-hydro-13-oxo-5*H*,13*H*,14*H*-[1]benzopyrano[3′,4′:5,6]pyrano[3,2-*e*][1,2,3,4]tetrazolo[1,5-*c*]pyrimidine **(4c)**

Brown solid, yield 0.49 g (53%); m.p. 187–189°C; IR (KBr, cm^−1^): 3387, 2987, 1711, 1658, 1589, 1366, 1267, 1129; ^1^H NMR (400 MHz, DMSO-*d*_6_): *δ* 4.83 (s, 1H, H-4), 6.22 (s, 1H, CH), 7.12–7.45 (m, 9H, ArH), 7.56 (d, 1H, ArH), 7.63 (t, 1H, ArH), 7.74 (t, 1H, ArH), 7.96 (d, 1H, ArH), 10.10 (s, 1H, NH); ^13^C NMR (100 MHz, DMSO-*d*_6_): *δ* 36.48, 63.76, 88.53, 103.74, 113.78, 119.94, 122.53, 124.64, 126.72, 127.39, 129.65, 130.48, 131.62, 132.73, 133.66, 139.12, 143.13, 153.70, 154.49, 157.11, 159.32, 160.53; MS *m/z*: 466 (M+1)^+^. Anal. Calcd for C_26_H_16_FN_5_O_3_: C, 67.09; H, 3.46; N, 15.05. Found: C, 67.18; H, 3.37; N, 15.10%.

### 4.19. 14-(4-Methylphenyl)-5-phenyl-6-hydro-13-oxo-5*H*,13*H*,14*H*-[1]benzopyrano[3′,4′:5,6]pyrano[3,2-*e*][1,2,3,4]tetrazolo[1,5-*c*]pyrimidine **(4d)**

White solid, yield 0.53 g (58%); m.p. 174–176°C; IR (KBr, cm^−1^): 3371, 2982, 1706, 1669, 1584, 1371, 1256, 1125; ^1^H NMR (400 MHz, DMSO-*d*_6_): *δ* 2.32 (s, 3H, CH_3_), 4.96 (s, 1H, H-4), 6.28 (s, 1H, CH), 7.08–7.40 (m, 9H, ArH), 7.46 (d, 1H, ArH), 7.56 (t, 1H, ArH), 7.71 (t, 1H, ArH), 7.93 (d, 1H, ArH), 10.18 (s, 1H, NH); ^13^C NMR (100 MHz, DMSO-*d*_6_): *δ* 20.96, 37.08, 64.16, 88.72, 103.32, 118.78, 119.72, 122.53, 125.36, 126.57, 128.71, 129.43, 130.53, 132.19, 133.58, 138.23, 139.21, 143.13, 153.70, 154.49, 157.11, 159.32, 160.57; MS *m/z*: 462 (M+1)^+^. Anal. Calcd for C_27_H_19_N_5_O_3_: C, 70.27; H, 4.15; N, 15.18. Found: C, 70.33; H, 4.24; N, 15.10%.

### 4.20. 14-(4-Methoxyphenyl)-5-phenyl-6-hydro-13-oxo-5*H*,13*H*,14*H*-[1]benzopyrano[3′,4′:5,6]pyrano[3,2-*e*][1,2,3,4]tetrazolo[1,5-*c*]pyrimidine **(4e)**

White solid, yield 0.57 g (60%); m.p. 165–168°C; IR (KBr, cm^−1^): 3388, 2997, 1701, 1665, 1591, 1360, 1267, 1129; ^1^H NMR (400 MHz, DMSO-*d*_6_): *δ* 3.85 (s, 3H, OCH_3_), 4.98 (s, 1H, H-4), 6.30 (s, 1H, CH), 7.01–7.44 (m, 9H, ArH), 7.50 (d, 1H, ArH), 7.54 (t, 1H, ArH), 7.73 (t, 1H, ArH), 7.84 (d, 1H, ArH), 10.14 (s, 1H, NH); ^13^C NMR (100 MHz, DMSO-*d*_6_): *δ* 37.43, 55.48, 64.34, 88.76, 104.07, 112.39, 114.73, 120.32, 123.59, 125.14, 126.37, 127.68, 129.38, 131.73, 133.81, 137.70, 138.32, 152.72, 154.68, 156.36, 158.42, 159.58, 160.53; MS *m/z*: 478 (M+1)^+^. Anal. Calcd for C_27_H_19_N_5_O_4_: C, 67.92; H, 4.01; N, 14.67. Found: C, 67.81; H, 4.06; N, 14.58%.

### 4.21. 14-(3-Nitrophenyl)-5-phenyl-6-hydro-13-oxo-5*H*,13*H*,14*H*-[1]benzopyrano[3′,4′:5,6]pyrano[3,2-*e*][1,2,3,4]tetrazolo[1,5-*c*]pyrimidine **(4f)**

Yellow solid, yield 0.50 g (51%); m.p. 191–194°C; IR (KBr, cm^−1^): 3399, 2982, 1718, 1659, 1582, 1368, 1251, 112; ^1^H NMR (400 MHz, DMSO-*d*_6_): *δ* 5.12 (s, 1H, H-4), 6.34 (s, 1H, CH), 7.11–7.38 (m, 5H, ArH), 7.46 (d, 1H, ArH), 7.53 (t, 1H, ArH), 7.60 (t, 1H, ArH), 7.76 (t, 1H, ArH), 7.88 (d, 1H, ArH), 7.94 (d, 1H, ArH), 8.12 (d, 1H, ArH), 8.20 (s, 1H, ArH) 10.24 (s, 1H, NH); ^13^C NMR (100 MHz, DMSO-*d*_6_): *δ* 37.82, 64.38, 88.83, 104.29, 113.68, 120.07, 123.33, 124.12, 125.57, 126.37, 127.41, 128.03, 131.32, 132.12, 133.73, 136.51, 138.56, 145.87, 148.42, 153.32, 154.66, 158.27, 159.87, 160.67; MS *m/z*: 493 (M+1)^+^. Anal. Calcd for C_26_H_16_N_6_O_5_: C, 63.41; H, 3.27; N, 17.07. Found: C, 63.48; H, 3.16; N, 17.16%.

### 4.22. General Procedure for the Synthesis of Fused Pyrano[3,2-*e*]tetrazolo[1,5-*c*][1,4]diazepines **(5a–f)**

A mixture of compound **2a–f** (2 mmol), 4-methoxyphenacyl bromide (0.45 g, 2 mmol), and sodium acetate (0.19 g, 2.4 mmol) in ethanol (15 mL) was refluxed for 16 h. After completion of the reaction (monitored by TLC), the reaction mixture was cooled to room temperature and then poured into water (30 mL). The solid separated was filtered, washed with water, dried, and purified by column chromatography on silica-gel using hexane/ethyl acetate (6 : 4) as eluent to obtain compound **5a–f**.

### 4.23. 15-Phenyl-6-(4-methoxyphenyl)-13-oxo-14*H*,15*H*-[1]benzopyrano[3′,4′:5,6]pyrano[3,2-*e*][1,2,3,4]tetrazolo[1,5-*c*][1,4]diazepine **(5a)**

White solid, yield 0.56 g (58%); m.p. 138–140°C; IR (KBr, cm^−1^): 2954, 1700, 1634, 1608, 1509, 1364, 1288, 1138, 1108; ^1^H NMR (400 MHz, DMSO-*d*_6_): *δ* 3.85 (s, 3H, OCH_3_), 4.58 (s, 1H, H-4), 6.13 (s, 2H, CH_2_), 7.10–7.15 (m, 2H, ArH), 7.28 (d, 2H, ArH), 7.37 (d, 2H, ArH), 7.43–7.53 (m, 3H, ArH), 7.70–7.77 (m, 2H, ArH), 7.82 (d, 1H, ArH), 7.93 (d, 1H, ArH); ^13^C NMR (100 MHz, DMSO-*d*_6_): *δ* 37.02, 48.43, 55.13, 103.90, 105.73, 113.01, 118.67, 119.21, 122.48, 124.61, 126.02, 126.25, 127.08, 128.22, 129.23, 132.85, 142.14, 152.14, 153.35, 157.22, 157.80, 159.53, 160.22, 161.87; MS *m/z*: 490 (M+1)^+^. Anal. Calcd for C_28_H_19_N_5_O_4_: C, 68.71; H, 3.91; N, 14.31. Found: C, 68.80; H, 3.83; N, 14.36%.

### 4.24. 15-(4-Chlorophenyll-6-(4-methoxyphenyl)-13-oxo-14*H*,15*H*-[1]benzopyrano[3′,4′:5,6]pyrano[3,2-*e*][1,2,3,4]tetrazolo[1,5-*c*][1,4]diazepine **(5b)**

White solid, yield 0.54 g (52%); m.p. 154–156°C; IR (KBr, cm^−1^): 2973, 1707, 1651, 1591, 1511, 1371, 1272, 1129, 1112; ^1^H NMR (400 MHz, DMSO-*d*_6_): *δ* 3.86 (s, 3H, OCH_3_), 4.56 (s, 1H, H-4), 6.17 (s, 2H, CH_2_), 6.98 (d, 2H, ArH), 7.29 (d, 2H, ArH), 7.44 (d, 1H, ArH), 7.56 (d, 2H, ArH), 7.61 (t, 1H, ArH), 7.72 (t, 1H, ArH), 7.84 (d, 2H, ArH) 7.98 (d, 1H, ArH); ^13^C NMR (100 MHz, DMSO-*d*_6_): *δ* 37.12, 48.51, 55.76, 104.23, 105.84, 113.14, 114.32, 119.71, 123.81, 124.93, 126.42, 128.72, 130.02, 130.86, 132.54, 133.67, 142.24, 152.28, 153.76, 156.92, 157.38, 159.63, 160.32, 161.78; MS *m/z*: 524 (M+1)^+^. Anal. Calcd for C_28_H_18_ClN_5_O_4_: C, 64.19; H, 3.46; N, 13.37. Found: C, 64.24; H, 3.57; N, 13.31%.

### 4.25. 15-(4-Fluorophenyl)-6-(4-methoxyphenyl)-13-oxo-14*H*,15*H*-[1]benzopyrano[3′,4′:5,6]pyrano[3,2-*e*][1,2,3,4]tetrazolo[1,5-*c*][1,4]diazepine **(5c)**

White solid, yield 0.51 g (51%); m.p. 173–176°C; IR (KBr, cm^−1^): 2989, 1704, 1648, 1606, 1512, 1371, 1266, 1134, 1104; ^1^H NMR (400 MHz, DMSO-*d*_6_): *δ* 3.86 (s, 3H, OCH_3_), 4.64 (s, 1H, H-4), 6.19 (s, 2H, CH_2_), 6.96 (d, 2H, ArH), 7.32 (d, 2H, ArH), 7.49 (d, 1H, ArH), 7.54 (d, 2H, ArH), 7.65 (t, 1H, ArH), 7.71 (t, 1H, ArH), 7.87 (d, 2H, ArH) 7.94 (d, 1H, ArH); ^13^C NMR (100 MHz, DMSO-*d*_6_): *δ* 37.43, 48.62, 55.81, 103.92, 105.74, 112.86, 114.36, 119.93, 123.25, 125.61, 126.32, 129.23, 130.13, 130.87, 132.85, 133.26, 143.17, 152.28, 153.70, 156.87, 157.47, 159.44, 160.53, 161.49; MS *m/z*: 508 (M+1)^+^. Anal. Calcd for C_28_H_18_FN_5_O_4_: C, 66.27; H, 3.58; N, 13.80. Found: C, 66.21; H, 3.67; N, 13.88%.

### 4.26. 15-(4-Methylphenyl)-6-(4-Methoxyphenyl)-13-oxo-14*H*,15*H*-[1]benzopyrano[3′,4′:5,6]pyrano[3,2-*e*][1,2,3,4]tetrazolo[1,5-*c*][1,4]diazepine **(5d)**

White solid, yield 0.55 g (55%); m.p. 165–168°C; IR (KBr, cm^−1^): 2983, 1709, 1641, 1614, 1511, 1360, 1273, 1131, 1109; ^1^H NMR (400 MHz, DMSO-*d*_6_): *δ* 2.32 (s, 3H, CH_3_), 3.89 (s, 3H, OCH_3_), 4.68 (s, 1H, H-4), 6.22 (s, 2H, CH_2_), 6.98 (d, 2H, ArH), 7.29–7.35 (m, 4H, ArH), 7.48 (d, 1H, ArH), 7.56 (t, 1H, ArH), 7.72 (t, 1H, ArH), 7.89 (d, 2H, ArH) 7.98 (d, 1H, ArH); ^13^C NMR (100 MHz, DMSO-*d*_6_): *δ* 20.94, 38.23, 48.71, 55.90, 103.53, 105.76, 114.47, 118.39, 119.68, 122.85, 125.83, 126.41, 129.26, 130.31, 130.72, 131.66, 133.53, 142.87, 153.12, 154.43, 156.93, 157.59, 159.52, 160.12, 161.23; MS *m/z*: 504 (M+1)^+^. Anal. Calcd for C_29_H_21_N_5_O_4_: C, 69.18; H, 4.20; N, 13.91. Found: C, 69.27; H, 4.25; N, 13.81%.

### 4.27. 6,15-Di-(4-methoxyphenyl)-13-oxo-14*H*,15*H*-[1]benzopyrano[3′,4′:5,6]pyrano[3,2-*e*][1,2,3,4]tetrazolo[1,5-*c*][1,4]diazepine **(5e)**

White solid, yield 0.59 g (57%); m.p. 201–203°C; IR (KBr, cm^−1^): 2991, 1711, 1667, 1600, 1521, 1371, 1265, 1142, 1101; ^1^H NMR (400 MHz, DMSO-*d*_6_): *δ* 3.84 (s, 3H, OCH_3_), 3.89 (s, 3H, OCH_3_), 4.77 (s, 1H, H-4), 6.28 (s, 2H, CH_2_), 6.98–7.10 (m, 4H, ArH), 7.39 (d, 2H, ArH), 7.48 (d, 1H, ArH), 7.54 (t, 1H, ArH), 7.70 (t, 1H, ArH), 7.81 (d, 2H, ArH) 7.93 (d, 1H, ArH); ^13^C NMR (100 MHz, DMSO-*d*_6_): *δ* 38.41, 48.83, 55.89, 55.96, 105.21, 105.93, 113.78, 114.82, 120.23, 123.71, 125.38, 127.08, 129.37, 130.43, 133.62, 136.81, 152.73, 153.88, 155.13, 156.22, 157.83, 158.34, 159.77, 160.43, 161.76; MS *m/z* 520 (M+1)^+^. Anal. Calcd for C_29_H_21_N_5_O_5_: C, 67.05; H, 4.07; N, 13.48. Found: C, 67.11; H, 4.14; N, 13.39%.

### 4.28. 15-(3-Nitrophenyl)-6-(4-methoxyphenyl)-13-oxo-14*H*,15*H*-[1]benzopyrano[3′,4′:5,6]pyrano[3,2-*e*][1,2,3,4]tetrazolo[1,5-*c*][1,4]diazepine **(5f)**

White solid, yield 0.51 g (48%); m.p. 228–230°C; IR (KBr, cm^−1^): 2987, 1708, 1658, 1603, 1501, 1376, 1263, 1127, 1118; ^1^H NMR (400 MHz, DMSO-*d*_6_): *δ* 3.89 (s, 3H, OCH_3_), 4.93 (s, 1H, H-4), 6.32 (s, 2H, CH_2_), 7.00 (d, 2H, ArH), 7.41 (d, 1H, ArH), 7.51 (t, 1H, ArH), 7.63 (t, 1H, ArH), 7.75 (t, 1H, ArH), 7.80 (d, 1H, ArH), 7.89 (d, 2H, ArH), 7.98 (d, 1H, ArH), 8.10 (d, 1H, ArH), 8.16 (s, 1H, ArH); ^13^C NMR (100 MHz, DMSO-*d*_6_): *δ* 38.71, 49.06, 55.98, 104.74, 105.91, 113.82, 114.67, 119.58, 123.62, 123.94, 124.21, 125.64, 126.91, 130.39, 131.13, 133.64, 135.67, 146.72, 148.31, 153.32, 154.66, 157.38, 158.32, 159.84, 160.77, 161.83; MS *m/z*: 535 (M+1)^+^. Anal. Calcd for C_28_H_18_N_6_O_6_: C, 62.92; H, 3.39; N, 15.72. Found: C, 62.81; H, 3.45; N, 15.62%.

## 5. Biological Protocol

### 5.1. Antimicrobial Activity

All of the newly synthesized compounds **2a–f**,** 3a–f**,** 4a–f**, and **5a–f** were screened for their antibacterial activities against the Gram-positive bacteria (*Bacillus subtilis* and* Staphylococcus aureus*) and the Gram-negative bacteria (*Pseudomonas aeuroginosa* and *Escherichia coli*). The antifungal activity of the compounds was assayed against *Candia albicans *and *Aspergillus niger*. The MICs of the compound assays were carried out using the microdilution susceptibility method. Ciprofloxacin was used as a reference antibacterial agent. Fluconazole was used as a reference antifungal agent. The test compounds, ciprofloxacin and fluconazole, were dissolved in DMSO at concentration of 800 *μ*g/mL, and they were then diluted in culture medium (nutrient agar for bacteria and Potato dextrose agar for fungi ), and two-fold serial dilution of the solution was prepared (400, 200, 100, 50, 12.5, and 6.25 *μ*g/mL). The tubes were incubated at 36°C for 24 h and 48 h for bacteria and fungi, respectively. The minimum inhibitory concentrations (MICs, *μ*g/mL) of the compounds were recorded as the lowest concentration of each chemical compound in the tubes with no turbidity (i.e., no growth) of inoculated bacteria/fungi.

## Figures and Tables

**Table 1:** Minimum inhibitory concentration (MIC, *μ*g/mL) of the synthesized compounds **2a–f**,** 3a–f**,** 4a–f**, and **5a–f**.

Compound	Gram-positive bacteria		Gram-negative bacteria		Fungi	
	*B. subtilis *	*S. aureus *	*P. aeuroginosa *	*E. coli *	*C. albicans *	*A*.* niger *
**2a**	400	400	—	—	400	400
**2b**	400	200	200	400	200	50
**2c**	400	400	400	200	50	400
**2d**	400	400	200	400	400	200
**2e**	200	400	400	200	200	400
**2f**	—	—	—	—	—	—
**3a**	400	400	200	200	400	200
**3b**	25	100	100	50	100	50
**3c**	200	400	200	400	200	200
**3d**	400	200	200	400	200	400
**3e**	25	100	100	25	50	100
**3f**	400	400	400	400	400	400
**4a**	400	400	400	400	200	400
**4b**	100	50	200	100	50	200
**4c**	400	200	400	400	400	400
**4d**	200	400	400	200	400	200
**4e**	200	100	50	100	200	50
**4f**	400	200	400	400	400	200
**5a**	—	—	—	—	—	—
**5b**	200	400	—	400	—	200
**5c**	—	400	400	—	400	400
**5d**	400	200	200	—	200	—
**5e**	200	400	200	400	200	400
**5f**	—	—	—	—	—	—
Ciprofloxacin	6.25	6.25	6.25	6.25		
Fluconazole					6.25	6.25
